# piRNAs in Gastric Cancer: A New Approach Towards Translational Research

**DOI:** 10.3390/ijms21062126

**Published:** 2020-03-19

**Authors:** Gleyce Fonseca Cabral, Jhully Azevedo dos Santos Pinheiro, Amanda Ferreira Vidal, Sidney Santos, Ândrea Ribeiro-dos-Santos

**Affiliations:** 1 Laboratório de Genética Humana e Médica, Programa de Pós-Graduação em Genética e Biologia Molecular, Universidade Federal do Pará, Belém 66.075-110, PA, Brazil; cabralffg@gmail.com (G.F.C.); jhully.as@hotmail.com (J.A.d.S.P.); amandaferreiravidal@gmail.com (A.F.V.); sidneysantos@ufpa.br (S.S.); 2 Programa de Pós-Graduacão em Oncologia e Ciências Médicas, Núcleo de Pesquisas em Oncologia, Universidade Federal do Pará, Belém 66.073-000, PA, Brazil

**Keywords:** piRNA, PIWI-interacting RNAs, gastric cancer, biomarkers, polymorphisms, INDEL, gene regulation, epigenetics, translational research, non-coding RNA biogenesis

## Abstract

Background: Gastric cancer is currently the third leading cause of cancer-related deaths worldwide, usually diagnosed at late stages. The development of new biomarkers to improve its prevention and patient management is critical for disease control. piRNAs are small regulatory RNAs important for gene silencing mechanisms, mainly associated with the silencing of transposable elements. piRNA pathways may also be involved in gene regulation and the deregulation of piRNAs may be an important factor in carcinogenic processes. Thus, several studies suggest piRNAs as potential cancer biomarkers. Translational studies suggest that piRNAs may regulate key genes and pathways associated with gastric cancer progression, though there is no functional annotation in piRNA databases. The impacts of genetic variants in piRNA genes and their influence in gastric cancer development remains elusive, highlighting the gap in piRNA regulatory mechanisms knowledge. Here, we discuss the current state of understanding of piRNA-mediated regulation and piRNA functions and suggest that genetic alterations in piRNA genes may affect their functionality, thus, it may be associated with gastric carcinogenesis. Conclusions: In the era of precision medicine, investigations about genetic and epigenetic mechanisms are essential to further comprehend gastric carcinogenesis and the role of piRNAs as potential biomarkers for translational research.

## 1. Introduction

Gastric cancer (GC) is the third leading cause of cancer-related deaths worldwide, responsible for 780,000 deaths in 2018 [1,2], thus, being a major public health issue. Gastric adenocarcinomas represent 95% of all GC cases and may be preceded by premalignant lesions that rarely present symptoms [3,4,5], leading to diagnosis at late stages, often with poor prognosis [6,7]. Hence, the development of tools to improve GC prevention and management is critical for disease control.

Proposed strategies to decrease disease burden are concentrated on its prevention [8,9], with the identification of high-risk groups, early diagnosis, and early patient management, including the treatment of premalignant lesions [8,10]. GC emerges from a complex interactive network of molecular alterations accumulated in the stomach tissue and environmental/behavioral risk factors that contribute to the disruption of cell homeostasis, allowing the acquirement of malignant features that (when not repaired) lead to tumor progression [4,11,12]. Consequently, molecular alterations associated with GC have been broadly explored, aiming for the development of novel disease-specific biomarkers and therapeutic targets [13,14].

Some of these molecular alterations are a consequence of somatic mutations, such as single nucleotide polymorphisms (SNPs) or insertion-deletion (INDELs), which are of special clinical interest due to their potential to disrupt gene functions [15,16,17]. For example, INDEL polymorphisms can cause genetic variations in piRNA sequences and change miRNA binding sites, increasing the susceptibility to various diseases, including gastric cancer [18,19,20]. Furthermore, the imbalance of epigenetic mechanisms, such as regulatory noncoding RNAs, has been associated with many types of cancer, affecting various regulatory pathways [18,21]. Among all noncoding RNAs, evidence suggests that piRNAs might have a role in gastric carcinogenesis and the deregulation of these molecules has been reported as a potential biomarker for GC prevention and management [22,23].

In this review, we discuss the current knowledge regarding piRNA-mediated regulation and piRNA functions. Also, we suggest that genetic alterations in piRNA genes may affect its biological roles and that the deregulation of these molecules might be associated with gastric carcinogenesis progress.

## 2. The piRNA Pathway

First described as Repeat Associated RNAs (rasiRNAs), PIWI-Interacting RNA—for short, piRNA—is a subclass of small noncoding RNAs discovered in developing *Drosophila melanogaster* [24] and renamed as piRNAs after the findings of their specific interactions with PIWI proteins [25,26,27].

Compared to microRNAs (the most well-established small noncoding RNAs) (Table 1), it is known that: (i) piRNAs have a Dicer-independent maturation process; (ii) they are generated from single-stranded precursors, (pre-piRNAs); and (iii) they are transcribed bidirectionally [28,29,30]. Among all noncoding RNAs, piRNAs are suggested as the most abundant and diverse small noncoding RNA, being derived from all types of genomic sequences [31], and more than 30,000 piRNA species were described in the human genome [32]. piRNA clusters can be inserted into both pseudogenes, intergenic, and protein coding-regions [33,34,35]. Moreover, although the main function of piRNAs is thought to be the silencing of transposable elements (TE) [28], there is a considerable number of piRNAs that derive from clusters depleted of TE in both protein-coding and intergenic regions with noncoding transcripts [33,34,36].

The presence of piRNAs in protein-coding regions suggested that these RNAs might be involved in the regulation of protein-coding mRNAs [37]. Also, since intergenic regions harbor several regulatory elements, such as long noncoding RNAs (lncRNA) [38,39], piRNAs could also regulate or be regulated by other noncoding molecules [35]. Hence, piRNA investigations are concentrated not only on developmental biology research, but also on their role in gene regulation and disease development.

**Figure 1:**
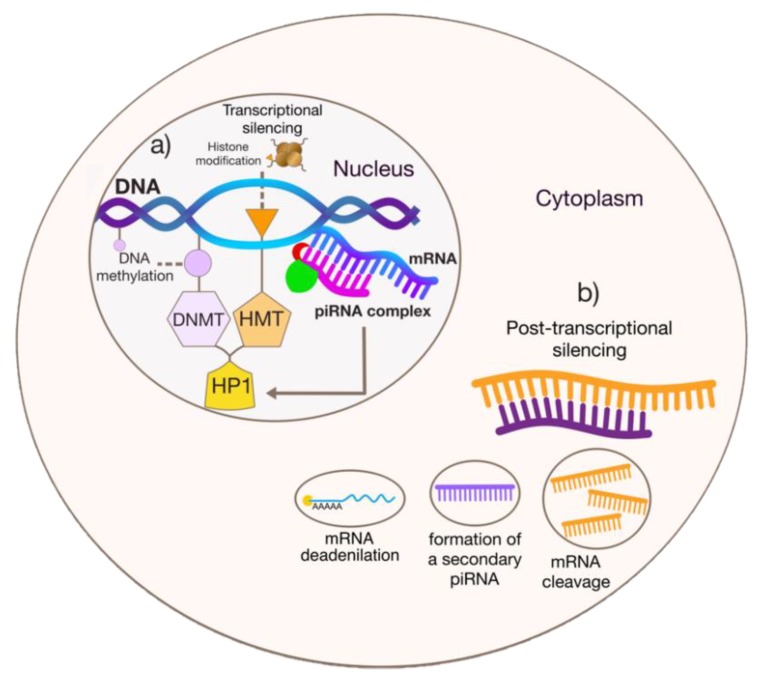
piRNA-mediated silencing. A major scheme showing piRNA silencing mechanisms. In (**a**), the translational silencing occurring into the nucleus. In this type of regulation, piRNAs mediate epigenetic changes in chromatin structure, mediating DNA methylation and/or histone modifications. In (**b**), the post-transcriptional silencing performed at the cytoplasm, in which piRNA recognizes a target mRNA and mediates its degradation by deadenylating or cleaving the mRNA.

It was demonstrated that the piRNA silencing complex can transcriptionally regulate gene functions through the interactions of Piwi with other proteins associated with chromatin remodeling, modifying the transcriptional status of the double helix. In *D. melanogaster* germ cells, when a piRNA recognizes a target, the complex cleaves the transcript of its target pre-mRNA and interacts directly with Heterochromatin Protein 1a (HP1a) [40,41]. This protein recognizes methylation residues in the ninth lysine of histone 3 (H3K9me) and mediates local gene silencing [42,43] (Figure 1).

HP1 can also mediate gene silencing by interacting with DNA methyltransferases. In murine germ cells, the PIWI–piRNA complex mediates the recruitment of HP1, triggering chromatin remodeling by DNA methylation [44,45,46] (Figure 1). DNA methylation is an epigenetic mechanism observed mainly in CpG sites in approximately 70% of the promoter regions of the human genome [47,48]. Whatever mechanism is used, it is already known that DNA methylation and histone modifications occur in an orchestrated, joint way where methylations influence histone modifications and vice-versa [46,49,50].

Both of these mechanisms occur during piRNA-mediated transcriptional silencing, which is linked with the piRNA primary pathway [28]. piRNA-mediated silencing can also occur at a post-transcriptional level, which in turn is linked to the secondary, loop-amplification pathway [29,51]. Although it has been intensely explored, the piRNA pathways are complex processes that are not fully elucidated and most of the available knowledge was obtained from studies in germ cells of model organisms, such as *D. melanogaster* and *Mus musculus*.

**Figure 2:**
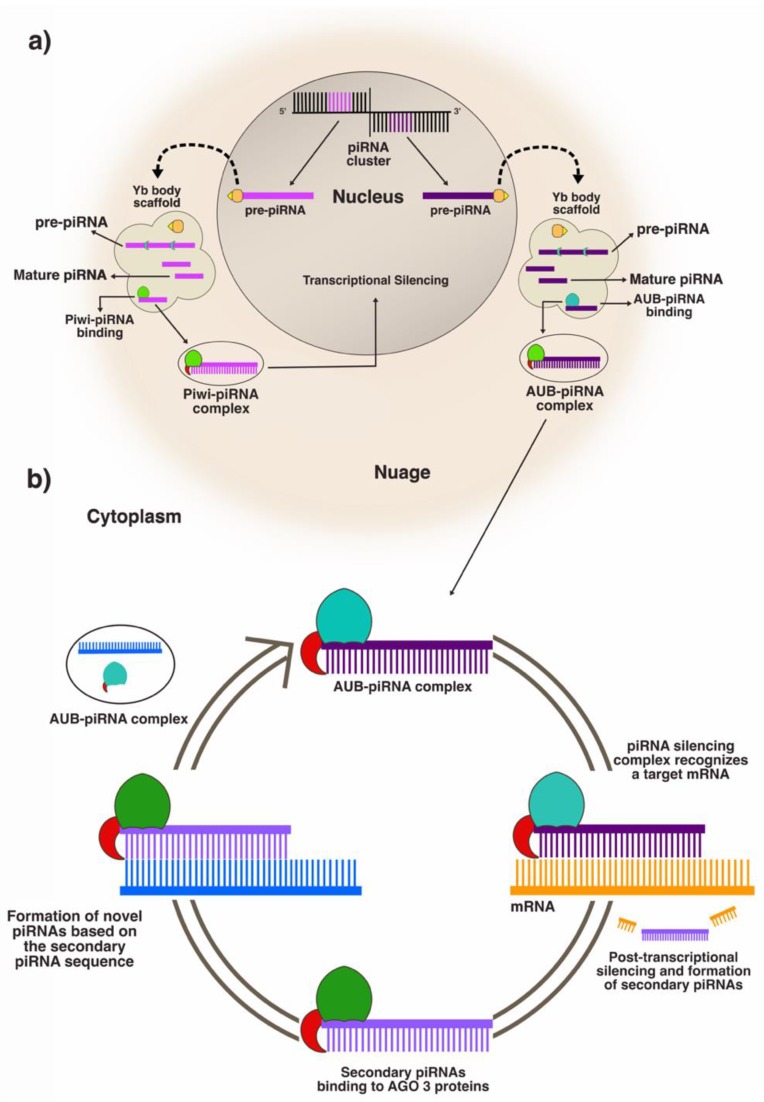
piRNA pathways and piRNA mediated silencing. In (**a**) is the primary pathway. The primary piRNA binds to Piwi protein to perform mRNA transcriptional silencing. In (**b**) is the secondary pathway. The primary piRNA binds to Aubergine (AUB), forming a complex that identifies and mediates mRNA post-transcriptional silencing. Secondary piRNAs are made during mRNA cleavage. These are used in new primary pre-piRNA maturation.

In summary, the primary pathway (Figure 2a) starts with the synthesis of pre-piRNA molecules in the nucleus, which is processed into mature piRNAs in the Nuage [52,53,54]. Then, the piRNAs bind to Piwi proteins, forming the piRNA silencing complex (piRISC), which returns to the nucleus where it performs transcriptional gene silencing [25,55,56]. PIWI is a subfamily of Argonaute proteins discovered in *Drosophila melanogaster*, composed by Piwi, Aubergine, and Argonaute 3 proteins (AGO3) [57,58], which are highly conserved [21] and expressed from pluripotent stem cells to differentiated somatic cells [59,60]. Transcriptional silencing (Figure 2b) starts when the PIWI–piRNA complex detects a complementary mRNA in the early stages of transcription, blocking accessibility of the translation machinery to the targeted sequence [61,62,63].

The secondary piRNA biogenesis pathway (also known as loop amplification or ping-pong pathway) (Figure 2b) is linked to the post-transcriptional silencing functions of the piRISC. Briefly, the mature piRNA binds to AUB, forming a complex that mediates the degradation of the mRNA, by deadenylating or cleaving a target mRNA in the cytosol [29]. This process also forms a secondary piRNA, which is matured and binds to AGO3 to form more piRNAs with sequences almost identical to the primary one [29,56,64].

Although several mechanistic features involving piRNA biogenesis and functions are not fully understood, it is suggested that mitochondrial proteins might be involved in piRNA maturation. For example, it was suggested that mitochondrial protein Zucchini (Zuc)—a phospholipase D (PLD) member that lies in the mitochondrial outer membrane and Nuage, is a conserved RNase essential for piRNA primary processing [65,66].

It has been demonstrated that Zuc mutant cells present less primary piRNAs [65,66,67]. Mutations in ZUC’s mammalian homolog, MitoPLD, lead to meiotic arrest, disruption of piRNA primary biogenesis, and de-methylation and de-repression of retrotransposons, leading to sterility [68]. Furthermore, other mitochondrial proteins were suggested as participants of piRNA biogenesis, such as Armitage, GPAT2, and PAPI, meaning many features of piRNA pathways remain unknown [69,70,71].

## 3. piRNA Functions

Differently from other noncoding RNAs, the functional roles of piRNA in cell homeostasis and disease development are poorly understood, especially in humans. In germ cells, piRNAs are important agents for the maintenance of genome integrity and the regulation of mRNA translation and stability [72,73], preventing DNA damage caused by TE in genomic sequences [27,74]. As described above, it is already known that piRNAs can transcriptionally regulate gene expression by inducing chromatin remodeling, repressing mRNAs harboring transposon sequences in the 3’UTR or 5’UTR regions [75].

Advances in molecular biology allowed further investigations regarding the putative functions of piRNAs in various eukaryotic organisms, which identified that piRNAs could also target non-TE sequences (such as mRNAs derived from protein-coding genes), modulating their expression levels [64]. In 2011, piR-015520 was the first human piRNA found regulating a protein-coding gene, and it was associated with the regulation of the Melatonin 1A receptor (MTNR1A) gene [37]. Other studies associated these molecules with the epigenetic regulation of genes involved in embryonic and gonadal development, sex determination, gametogenesis, apoptosis, and stem cell division [21,76,77,78,79].

At the somatic level, piRNAs were found to be expressed not only in somatic cells, but also in body fluids [80,81,82,83]. However, piRNA functions in the soma remain elusive, due to the lack of information on functional assays involving piRNAs. Studies suggest that piRNAs may be involved in the epigenetic regulation of genes related to neurogenesis, neuronal activity, and plasticity [45,84,85] and that piRNAs derived from pseudogenes may regulate parental genes [86]. It was also suggested that antisense piRNAs may regulate immune response and self-tolerance genes [87]. Moreover, a small-nucleolar-RNA-derived piRNA (sno-piRNA) was demonstrated to be present in primary CD4 + T-lymphocytes, which prevented their differentiation into Th2 T-cells by regulating interleukin-4 (IL-4) levels [88].

Additionally, piRNAs can also be expressed in mitochondria, derived from sequences of tRNA, 12S, and 16S rRNA, and mitochondrial protein-coding genes, such as COX2, ND4L, and ND5 [89], suggesting a possible cross-talk between mitochondrial and nuclear piRNA transcripts and an involvement of mt-piRNAs in bioenergetics and cellular response to oxidative stress. As the mitochondria is an organelle involved in several cellular critical processes, including bioenergetics and apoptosis, functional studies are necessary to better understand the role of piRNAs in mitochondrial epigenetics and how it affects health and disease development.

The functionality of the piRNAs is associated with the expression of Piwi-like (PIWIL) genes, that produce PIWIL proteins, essential for piRNA biogenesis [21]. The Piwi subfamily is involved in stem cell maintenance and renewal and is associated with tissue regeneration and cell differentiation [21,90,91]. The human genome encodes four PIWIL genes, known as PIWIL1 (HIWI), PIWIL2 (HILI), PIWIL3 (HIWI3), and PIWIL4 (HIWI2); some of which were found to be highly expressed in several human cancers [92,93,94,95,96].

Studies have demonstrated that the piRNA-PIWI pathway has a role in the translation machinery. For example, PIWIL4 protein, which resides strictly in the cytoplasm, is associated with the translation of ribosomes and other proteins involved in translational machinery, and evidence suggests that somatic piRNA regulation functions may precede transposon silencing [97]. Furthermore, it was observed that piRNAs can also regulate gene expression in a miRNA-like manner, post-transcriptionally inhibiting mRNA translation by incomplete base pairing [98,99].

All this knowledge leads to the suggestion that the deregulation of elements involved in the piRNA silencing complex may have a role in tumorigenesis [64,100,101]. As both cancer and stem cells are capable of self-renewal [102], the imbalance in piRNA expression levels may lead to increased silencing of genomic regions, resulting in a “stem-like” state in which several genomic regions become methylated, including sequences harboring tumor suppressor genes and cell cycle negative regulators, thus driving cancer progression [101].

Recent studies include the investigation of potential interactions between piRNAs and other noncoding RNAs, leading to the hypothesis of a broad regulatory network involving several epigenetic mechanisms. For instance, it was reported that piRNAs are capable of mediating the degradation of mRNAs and lncRNAs in murine germ cells [35]. Since lncRNAs have been associated with metastasis [103], it is necessary to understand the interplay among regulatory RNAs and how these interactions are involved in tumor invasiveness and metastasis.

Hence, the identification cancer-associated piRNAs and the functional analysis of piRNA target genes may contribute to preventive and therapeutic strategies against cancer [102]. Recent studies have associated the differential expression of piRNAs to various cancers, suggesting that changes in the expression levels of somatic and mitochondrial piRNAs may deregulate important genes involved in cell metabolism and cancer hallmark pathways [23,100,104,105]. More recently, studies have also investigated polymorphisms in small RNAs, including miRNAs and piRNAs, revealing that these variants may affect susceptibility risk to cancer progression [19,20,106].

## 4. The piRNA Pathway in Gastric Cancer

### 4.1. Differential Expression of piRNAs in Gastric Cancer

Several studies have demonstrated that piRNA expression profiles can distinguish tumor tissue from a non-cancer one, suggesting piRNAs for further research on cancer biomarkers. Although piRNAs have been broadly explored over the last decade, few studies investigated the role of piRNAs in gastric carcinogenesis. The first investigation on piRNA expression profile in cancer cells evaluated their expression in tissue of colon, lung, breast, and gastric cancer and human cell lineages of GES-1 (human gastric epithelial cells), HepG2 (hepatic carcinoma), HeLa (cervical cancer), Bcap-37 (breast cancer), MSTO-211H (mesothelioma), NCI-H446 (lung cancer), MGC-803, and SGC-7901 (gastric cancer) [102]. This study revealed the overexpression of piR_651 (GenBank: *Homo sapiens* piR_30675) in human GC tissue and cell lines and that the levels of piR_651 were higher in individuals with advanced GC stages when compared with those in earlier stages. Furthermore, the inhibition of piR_651 restrained cell proliferation in MGC-803 and SGC-7901 cell lines. The up-regulation of piR_651 was also detected in colon, lung, and breast cancer tissues and in HeLa, Bcap-37, MSTO-211H, and NCI-H446 cell lines.

**Figure 3:**
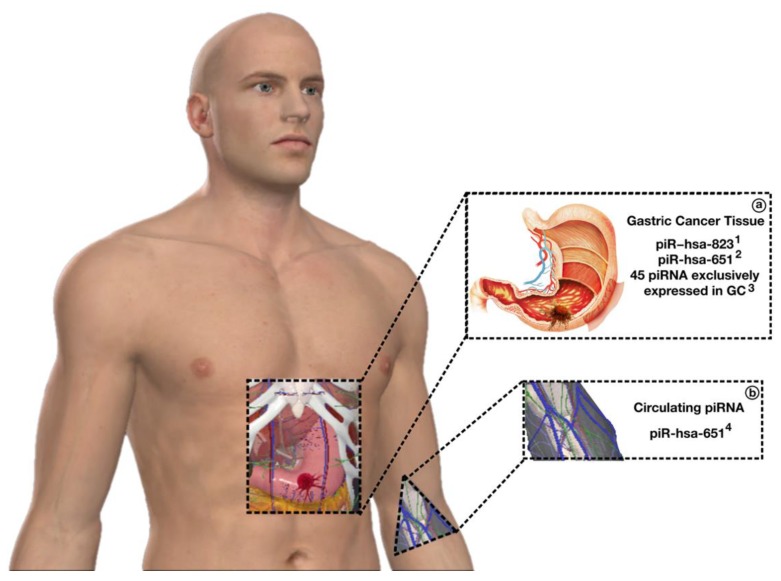
piRNAs in gastric cancer. To date, various studies have reported several differentially expressed piRNAs in gastric cancer patients, both in cancer tissue (**a**) and in the bloodstream (**b**). The presence of deregulated piRNAs in stomach cancer patients demonstrates the potential of these molecules of being used as gastric cancer biomarkers.

Another piRNA, piR-823 (GenBank: *Homo sapiens* piR_30847), was found to be downregulated in GC cells [107]. Downregulation of piR_823 increased the susceptibility to multiple myeloma progression and its overexpression was associated with increased cell proliferation and inhibited apoptosis in colorectal cancer cells [104,107], indicating tissue-specific piRNA levels. Further analysis in GC cell lines showed that the normalization of piRNA levels was able to inhibit GC cell proliferation, as both the inhibition of piR_651 and the use of mimics of piR_823 had that effect both in vitro and in vivo [102,107]. Furthermore, piR_651 was found circulating in the peripheral blood of GC patients (Figure 3) [108]. Thus, these piRNAs were proposed as potential biomarkers of GC progression [107].

Advances in molecular research and high-throughput sequencing technologies allowed more detailed analyses of piRNA expression in non-malignant and tumor cells. piRNA transcriptome assays using samples of 12 different cancers, including gastric cancer, revealed that both non-malignant and tumor cells expressed hundreds of piRNAs in tissue-specific and tumor-specific patterns [100]. Results indicate that piRNAs may have tissue-specific functions and that the deregulation of piRNAs have different effects depending on where it occurs. In summary, non-cancer tissues presented lower piRNA expression levels, varying between 29–30 nucleotides long and homogenous tissue-specific expression patterns (although there were those with highly heterogeneous profiles, as observed in stomach samples). Adversely, piRNA expression in tumoral tissues was overall up-regulated in all tumor samples, being grouped in tissue-specific piRNAs with shorter sequences (24–28 nucleotides long), and piRNAs expressed in several cancers (pan-cancer piRNAs), in which sequences were longer than those for non-malignant piRNAs.

These observations indicated that (i) distinct piRNA profile patterns are associated with specific tissues, suggesting tissue-specific functions; (ii) piRNA was found in all cancer samples, indicating their potential involvement in the regulation of key carcinogenic processes; (iii) somatic and mitochondrial piRNAs can be associated with key tumor clinicopathological features, being able to indicate patient prognosis; and (iv) mitochondrial piRNAs found up-regulated in almost all tumor samples suggest the participation of a subset of piRNAs in cancer metabolism [100]. These findings support the hypothesis that piRNAs are important molecules that regulate key processes (such as cell metabolism and cancer hallmarks pathways) and when imbalanced may promote tumor progression.

In the context of GC, these findings allowed the development of an atlas of differentially expressed (DE) piRNAs in GC using data from The Cancer Genome Atlas (TCGA) cohort, revealing 312 piRNAs in non-cancer and cancer samples, half of which were found deregulated, and most of these were overexpressed in GC samples [23]. The atlas also showed a group of piRNAs expressed in tissue-specific and GC-specific manners and that 70% of all expressed piRNAs derived from protein-coding regions. However, since piRNA’s functions in the soma were not established, the possible roles of piRNAs in the regulation of non-cancer stomach and GC tissues remain elusive. Their importance in both normal and GC contexts is undeniable, though. Additionally, the identification of piRNAs associated with recurrence-free survival in GC patients [23] highlights the potential utility of these molecules in a translational context.

Overall, several studies have shown clinical relevance of piRNAs in gastric cancer and other cancers, emphasizing their potential for use as cancer biomarkers and/or therapeutic tools, since the deregulation of piRNAs has been related to inhibition of cell-cycle arrest and apoptotic signals, and promotion of cell proliferation, tumor invasiveness, and metastasis [23,102,107,109].

Despite the broad implications of piRNA-mediated epigenetic regulation for cancer research, virtually nothing is known regarding the impact of genetic alterations within piRNA genes—mainly polymorphisms—in a carcinogenic context. It is suggested that certain piRNAs may possess multiple gene targets and changes on its functions can have a significant impact on multiple cancer-relevant pathways.

### 4.2. piRNAs Polymorphisms in Gastric Cancer

According to ENCODE data, most of the human genome is composed of non-coding sequences and alterations in these segments may have a significant impact on gene regulation [110,111]. Genetic variants were identified in several genes involved in cancer-related pathways, directly impacting tumor progression [112]. INDEL and SNP polymorphisms were highlighted as gene function modifiers in multiple studies investigating cancer susceptibility, such as gastric, breast, and colorectal tumors, among others [113,114,115,116,117].

Several studies report INDEL mutations associated with gastric cancer [13,118,119]. The majority of these studies investigate the role of genetic variants in protein-coding genes, however few studies evaluated the impact of polymorphisms in noncoding RNAs [120]. For example, some reports associated INDEL mutations at miRNA binding sites in genes related to gastric cancer and other tumors, as reported by Gao et al., who observed an indel polymorphism within the 3’UTR region of the gene (which is associated with liver cancer in Chinese population) that affect the binding of miR-122 and miR-378, influencing the regulation of interleukin 1 alpha (IL-1a) by these miRNAs [121]. Moreover, another study suggested three miRNA binding site SNPs in TYMS 3’UTR that were associated with an increased risk and poor survival in gastric cancer [106].

Studies with piRNAs can help elucidate how genetic variants affect regulatory processes and disease development. However, there is a lack of information about the impact of these variants in piRNA functions and how this can influence carcinogenic processes involving piRNAs. Until the moment of this review, no study involving INDEL variants in gastric cancer was published, although recent studies have associated some SNP variants in piRNA sequences with increased risk of cancer development in various cancers, such as breast cancer, glioma, and melanoma [19,20,122].

**Figure 4:**
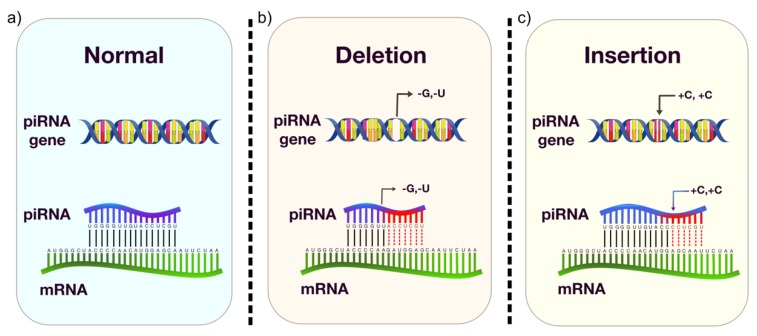
Potential effects of insertion-deletion (INDEL) polymorphisms in piRNA. piRNA normal regulation (**a**) is sequence-dependent, by perfect or imperfect complementarity. When a deletion (**b**) or an insertion (**c**) occurs, the alteration changes the molecule’s structure. Hence, even if piRNA can regulate by imperfect complementarity, structural alterations caused by INDEL variants may disrupt their functions, affecting the regulation of several genes.

Analyses in breast cancer tissue and cell lines revealed that polymorphisms within functional piRNA clusters may alter the functions of the mature piRNAs, leading to their deregulation, which is associated with the carcinogenic process [19,100]. A study performed in vitro functional analyses to investigate the functional impact of the piRNAs and its variant allele in glioma cell lines, indicating the variant rs147061479 in piR-598 as associated to increased glioma risk [122]. The treatment with mimics of the wild-type piRNA significantly diminished cell viability, while the addition of mimics of the alternative piRNA eliminated the antiproliferative effect of piR-598 and promoted colony formation. Additionally, PIWI/piRNA SNPs were associated with disease outcome, as shown by Zhang et al. [20], who found the association between the PIWI/piRNA gene DCP1A/rs11551405 with increased risk of melanoma disease-specific death in both discovery and validation datasets. In silico analyses showed that this variation may be located in miRNA binding sites. This suggests potential crosstalks among regulatory RNAs, such as miRNAs and piRNAs. Furthermore, as shown in miRNA binding sites, INDEL polymorphisms could also have an impact on piRNA functions affecting piRNA-target binding, contributing to gastric cancer susceptibility (Figure 4).

INDEL polymorphisms, which give rise to frameshifts, could have more impact than SNPs because of third base degeneracy, and the former is more likely than the latter to moderate the structural function of regulatory elements, contributing to phenotypic differences in humans, including susceptibility to diseases [123,124]. Since piRNA functions and their role in carcinogenesis remain unclear, it is important to investigate both genetic and epigenetic aspects involving these molecules to further understand their role in healthy and tumoral processes. Nevertheless, although research on the role of piRNAs in the tumorigenesis of GC and other cancers is still in its first steps, it is an emerging area that can contribute to the understanding of molecular processes associated with cancer.

## 5. Future Perspectives

Recent studies on gastric cancer research have highlighted the importance of epigenetic processes in cellular homeostasis and tumorigenesis. Since the early 2000s, the role of several noncoding RNAs in gastric carcinogenesis has been investigated revealing various mechanisms by which these molecules regulate key genes for cellular homeostasis, and how the disruption of these mechanisms may lead to cancer progression.

Since its discovery, a lot of knowledge has been collected about piRNA pathways in critical cellular processes, both in the germline and somatic cells, confirming their relevance in health and cancer development, and studies on gastric cancer epigenetics have confirmed the importance of these molecules in tumor progression. Although the role of piRNAs in GC’s development remains unclear, reports reveal differences in piRNA expression between tumoral and non-tumoral cells, suggesting their potential for acting as disease biomarkers.

High-throughput sequencing is currently being used to reveal novel piRNAs and known isoforms in several cancers. However, further investigations are necessary to better understand the functional and mechanistic features involved in piRNA pathways, such as putative target genes. For instance, in vitro and in vivo functional assays could be performed to investigate how piRNAs regulate genes and interfere with their function. Potential interactions between piRNAs and other epigenetic molecules, such as miRNA or other proteins involved in chromatin remodeling, can also be explored both in silico and in protein immunoprecipitation sequencing assays. In silico functional enrichment and expression quantitative trait loci (eQTL) analyses could be performed to explore both gene–piRNA interactions and the interplay between noncoding RNAs and target genes.

As discussed above, there is a difference in piRNA length comparing normal and tumoral tissues. This phenomenon could be explained by the presence of INDEL polymorphisms within piRNA sequences, which could interfere with piRNA’s function, leading to the disruption of piRNA-mediated regulation. Thus, considering the relevance of genetic variants in cancer research and the evidence of genetic variants within piRNA genes, it is important to investigate the impact of piRNA polymorphisms. These variants can be identified through genomic analyses, such as DNA sequencing, and bioinformatic analyses can be performed to evaluate the functional alterations. These studies may help elucidate the mechanistic features of piRNA that may be associated with carcinogenesis.

Furthermore, as previously reported, piRNAs associated with cancer progression can be found circulating in patients’ bloodstream, but it was not described how piRNA becomes circulating, which is important since circulating biomarkers are preferred for being less invasive. In a translational approach, validation studies evaluating piRNA copy number and relative expression by qPCR assays could be developed aiming to bring these research findings into the medical routine.

Ultimately, to the best of our knowledge, there are no ongoing or published clinical trials involving piRNAs. Hence, it is essential to further investigate the involvement of these molecules in gastric cancer-related pathways, since it can reveal novel disease biomarkers and/or anticancer therapy targets that could be used in clinical practices such as screening, diagnosis and treatment.

## 6. Conclusions

In this review, we summarized the main aspects of piRNA-mediated epigenetic regulation, highlighting the relevance of these molecules for the maintenance of essential cellular processes, and their potential influences on health and cancer progression. In the era of precision medicine, investigations about genetic and epigenetic mechanisms associated with cancer are essential to further comprehend gastric carcinogenesis aiming to improve current clinical practices and development of new anticancer approaches.

Gastric cancer remains one of the most lethal cancers, currently being a public health issue, with more than a million cases and 700,000 deaths worldwide. Prevention remains the best way to decrease its burden, and effective strategies include the development of tools for identification of high-risk groups, treatment of precancerous lesions, and early diagnosis. Thus, it is important to investigate all molecular aspects involving gastric cancer’s progression, aiming to find new tools for prevention and patient management. In this context, piRNAs have been demonstrated as potential tools for gastric cancer translational research.

## Figures and Tables

**Table 1:** Comparison between miRNA and piRNA’s main features.

Features	miRNA	piRNA
Subclass	Small noncoding RNA	Small noncoding RNA
Length	~21	~31
Precursor	Double-stranded, hairpin RNA	Single-stranded RNA
Complexity	>2000 known in humans	>30,000 known piRNAs
Genomic Annotation	Noncoding regions, coding genes	Transposable elements, noncoding regions, and coding genes
Maturation	Dependent on Dicer	Independent of Dicer
Target	mRNA	mRNA
Function	mRNA repression	mRNA and transposon repression, DNA methylation, histone modification
Associated Proteins	AGO2	PIWI/PIWI-Like
Silencing Mechanism	Post-transcriptional	Transcriptional and post-transcriptional

## Abbreviations

lncRNA	Long Non-coding RNA
INDEL	Insertion-Deletion Polymorphisms
MDPI	Multidisciplinary Digital Publishing Institute
DOAJ	Directory of open access journals
eQTL	Expression Quantitative Trait Loci
mRNA	Messenger RNA
piRNA	Piwi-Interacting RNA
rRNA	Ribosomal RNA
SNP	Single Nucleotide Polymorphism
tRNA	Transfer RNA
UTR	Untranslated Region
GC	Gastric Cancer
